# Colovesical Fistula and Amyloidosis

**DOI:** 10.7759/cureus.80695

**Published:** 2025-03-17

**Authors:** Samantha Leng, Wei Ming Ong, Reizal Mohd Rosli, Tarini Fernando, Vinna An

**Affiliations:** 1 Colorectal Surgery, Eastern Health, Melbourne, AUS; 2 Pathology, Eastern Health, Melbourne, AUS

**Keywords:** gastrointestinal amyloid, hartmann's procedure, immunoglobulin light chain amyloidosis, systemic amyloidosis, vesicocolic fistula

## Abstract

Systemic amyloidosis results from an abnormal deposition of toxic insoluble beta-sheet fibrillar protein in extracellular tissues, causing damage to multiple organ systems. Amyloid proteins (e.g., transthyretin, light chains, and serum amyloid A) may infiltrate the mucosa or vascular structures, resulting in gastrointestinal manifestations, including bleeding and diarrhoea. We discuss a case of a 71-year-old male with systemic immunoglobulin G kappa amyloid light chain amyloidosis who developed a colovesical fistula related to amyloid deposition.

## Introduction

Systemic amyloidosis is a disorder of abnormal deposition of beta-sheet fibrillar protein aggregates in various tissues remote to sites of amyloid protein precursor production [1,2]. Although there is a broad spectrum of possible aetiologies of systemic amyloidosis, amyloid light chain (AL) disease is the most common in the Western world [1,2]. Less common subtypes include familial transthyretin-associated amyloidosis (ATTR), hereditary, and age-related wild-type ATTR amyloidosis [3]. Systemic AL amyloidosis is a heterogenous disease that is known to infiltrate all organ systems except for the brain, resulting in clinical manifestations such as renal impairment, restrictive cardiomyopathy, peripheral neuropathy, pseudohypertrophy of skeletal muscle, gastrointestinal dysfunction, or macroglossia [1]. The pathophysiological mechanism of systemic amyloidosis is the deposition of insoluble amyloid precursor protein fibrils (e.g., transthyretin, light chains, and serum amyloid A) in extracellular tissue eventuating in alteration of tissue architecture, inflammation oxidative stress, and subsequent apoptosis activation [3]. When amyloid proteins are deposited into the mucosal or neuromuscular layers of the gastrointestinal tract, patients may present with gastrointestinal bleeding, malabsorption, protein-losing gastroenteropathy, or chronic gastrointestinal dysmotility [2]. Previously documented surgical complications that have occurred secondary to systemic amyloidosis include pseudo-obstruction, intussusception, obstructing colonic mass, and gastrocolic fistula [4-7]. To our knowledge, there have been no documented cases of colovesical fistulae associated with amyloidosis thus far. We present a case of a patient with a history of systemic AL amyloidosis with colovesical fistula requiring operative management.

## Case presentation

A 71-year-old male presented to the emergency department of a metropolitan tertiary hospital in Melbourne, Australia, with recurrent, painless per rectal bleeding. He had a history of immunoglobulin G (IgG) kappa systemic AL amyloidosis. This was diagnosed six years prior with cardiac manifestations of the disease, which were dyspnoea, orthopnoea, and paroxysmal nocturnal dyspnoea. He was undergoing active chemotherapy at the time of his presentation to the hospital, having previously had an unsuccessful autologous stem cell transplant. In addition, this patient had amyloidosis-related end-stage kidney disease, as well as atrial fibrillation, sick sinus syndrome, hypertension, and obstructive sleep apnoea. He reported suffering from chronic diarrhoea without a clear cause, which had been previously investigated. To note, he had no previous clinical history of diverticulitis.

**Figure 1:**
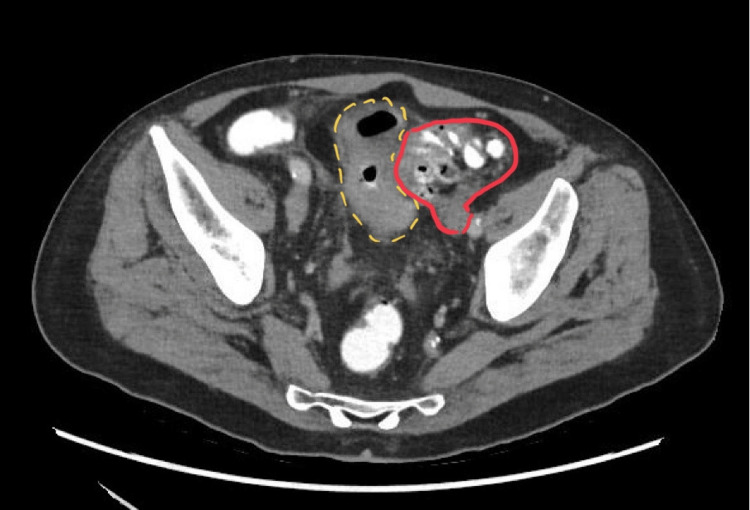
Axial slice computed tomography angiogram image demonstrating colovesical fistula. The bladder (with gas noted within it) is outlined by the yellow hatched line, and the sigmoid colon is outlined in red.

The patient had a gastroscopy and colonoscopy three months prior to investigate the rectal bleeding, which showed only mild gastritis and sigmoid diverticulosis, but no actual bleeding source was found. In this current presentation, a computed tomography (CT) angiogram did not demonstrate active contrast extravasation. However, a complex fistula was noted involving the sigmoid colon, ileum, and bladder dome, with an associated 28 x 28 mm collection abutting the bladder dome. This is demonstrated in the CT image in Figure 1. He was treated conservatively with intravenous antibiotics and was followed up in the colorectal outpatient clinic. Following his clinic appointment, he was planned for a cystoscopy to interrogate the location and nature of the fistulous opening for surgical planning.

**Figure 2:**
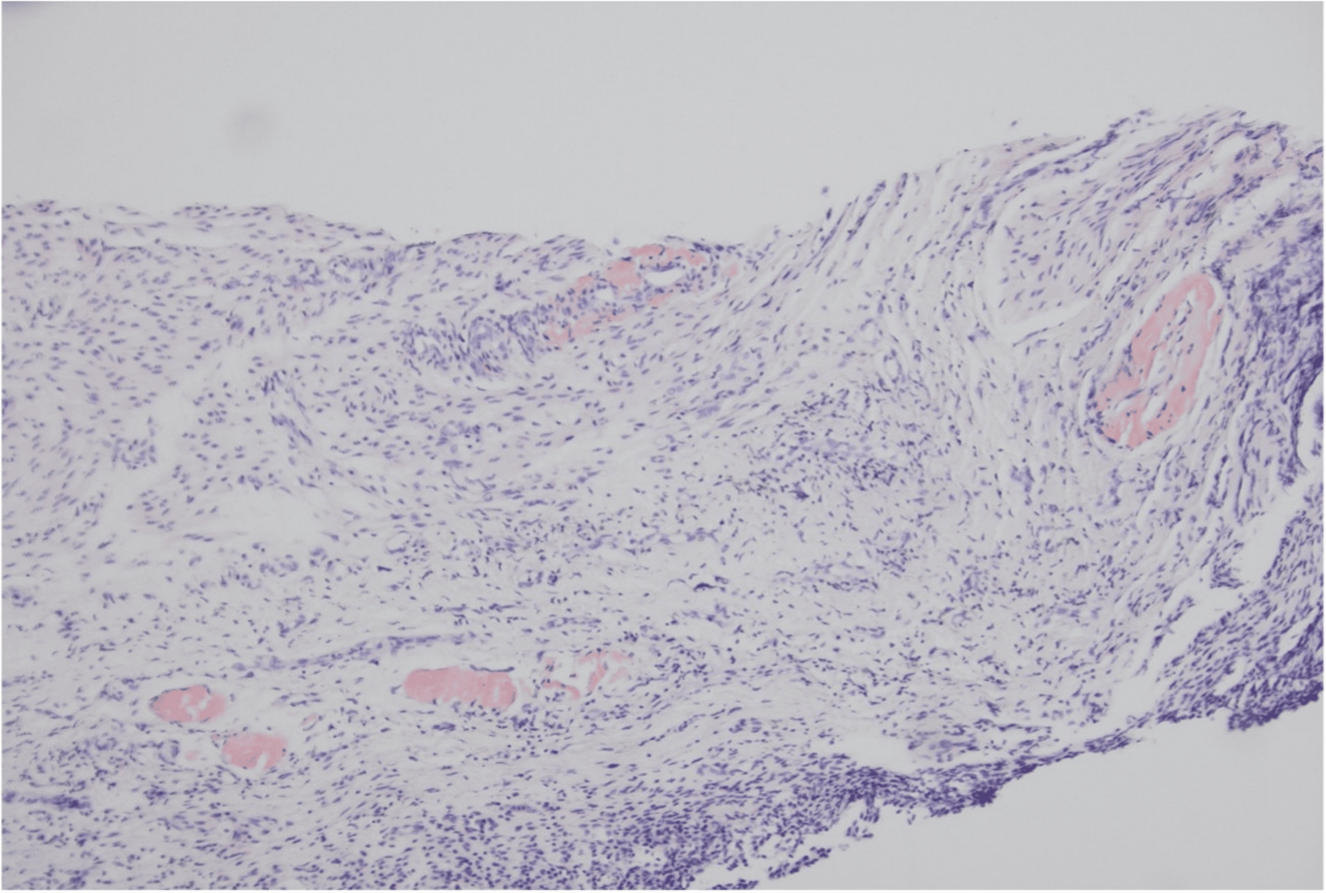
Histology slide with Congo red stain demonstrating amyloid in the vessel walls within the bladder biopsy.

**Figure 3:**
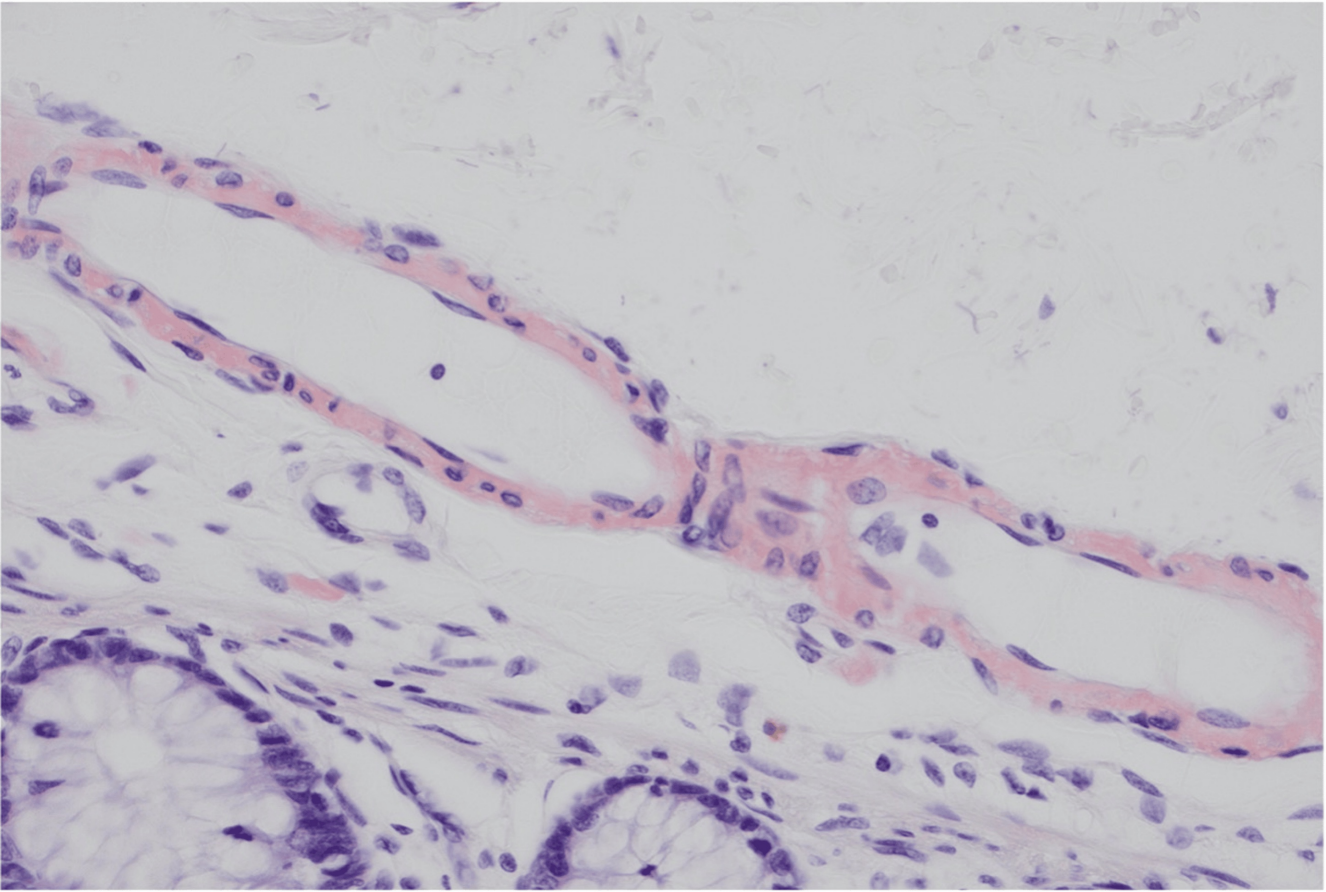
Histology slide with Congo red stain demonstrating amyloid infiltration into the vessel walls.

**Figure 4:**
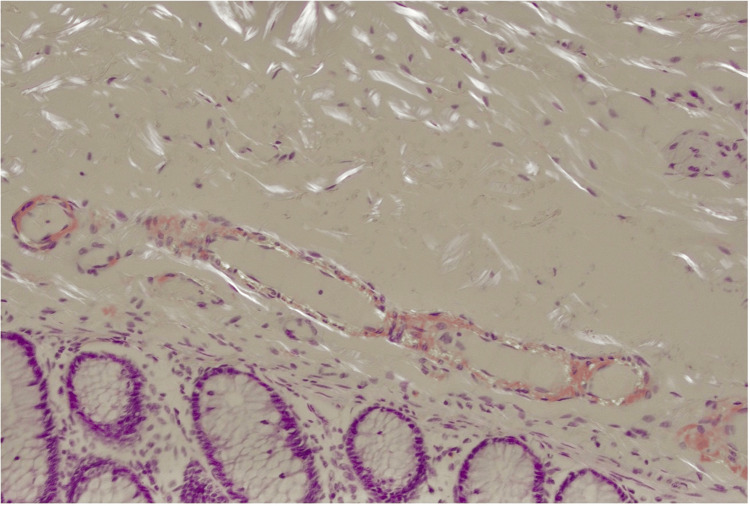
Histology slide with apple-green birefringence demonstrating amyloid in the vessel walls within the bowel specimen. The original histological image has been edited to increase brightness and reduce shadow for the purpose of image clarity.

The patient underwent cystoscopy, which demonstrated an apparent fistulous tract in the midline of the posterior wall of the bladder, with surrounding appearances of oedema and inflammation, as well as active pus discharging from the central sinus. A biopsy of the bladder wall was taken during the cystoscopy at the level of the fistula tract. Following this, the patient underwent an uncomplicated laparoscopic Hartmann’s procedure and primary repair of the bladder wall defect. Histological assessment of the bladder biopsy returned positive for reactive urothelial changes indicative of inflammation and amyloid deposition within the vasculature (Figure 2). The colonic segment demonstrated the prominent involvement of amyloid in submucosal blood vessels and within the muscularis propria, as well as diverticula (Figures 3, 4). Amyloid deposition is identified as staining red on Congo red stain, and when viewed under polarized light, they exhibit a characteristic "apple-green" birefringence. Neither the bladder biopsy nor the colonic segment displayed any evidence of dysplasia or malignancy. The patient’s postoperative recovery was complicated by a urinary tract infection (UTI) associated bacteraemia. He was discharged 14 days following his operation.

## Discussion

The pathophysiological mechanisms by which gastrointestinal amyloidosis occurs are thought to be the infiltration of amyloid precursor proteins into layers of the gastrointestinal tract [2]. AL amyloid disease, in particular, is more commonly found in the muscularis mucosa, submucosa, and muscularis propria, forming protrusions of tissue and possibly leading to symptoms of bowel obstruction [2]. Mucosal amyloid infiltration can also result in increased tissue friability and erosions, which presents symptomatically as diarrhoea or features of malabsorption [1,2]. This, along with vascular friability and bowel ischaemia, can often present clinically as gastrointestinal bleeding [2]. Additionally, amyloidosis is known to cause neuromuscular infiltration of the gastrointestinal tract, resulting in dysmotility effects that can manifest as dysphagia, gastroparesis, generalised bloating, or colonic pseudo-obstruction [1,2].

Colovesical fistulas have three common aetiologies: diverticular disease, malignancy, and inflammatory bowel disease [8,9]. In the case of diverticular disease, it is thought that peristalsis increases intraluminal pressure within the diverticulum, causing macro- and microscopic perforations, which lead to diverticular abscess or phlegmon that can rupture into the adjacent bladder to create a fistulous connection [8,9]. In malignancy, the abnormal connection between bowel and bladder epithelial surfaces is a result of direct invasion of carcinoma, and in inflammatory bowel disease, the pathogenesis is likely related to long-standing transmural inflammation [8]. We acknowledge that the patient we described was found to have diverticular disease on histopathological assessment. However, our patient had no previous documented episodes of diverticulitis and no history of diverticular abscess or phlegmon that subsequently may have resulted in the eventual formation of his colovesical fistula.

A prior case study from 2002 describes a 64-year-old male with a gastro-colic fistula and systemic amyloidosis [7]. In this case, a post-mortem evaluation of the patient demonstrated that the fistula had likely developed secondary to microvascular insufficiency, leading to localised ischaemia. This hypothesis was supported by the presence of extensive perivascular amyloid deposits [7]. Our patient shared similar demographics but was undergoing treatment for amyloidosis. Prominent amyloidosis was seen invading the vasculature of both the colonic segment and the bladder wall fistulous tract on the histology. We hypothesise that the amyloid deposition contributed to local ischaemia and connective tissue degradation, predisposing the sigmoid colon to forming a fistulous formation as the patient never experienced symptoms in keeping with diverticulitis or diverticular perforation before the identification of a colovesical fistula.

## Conclusions

While systemic amyloidosis can manifest in multi-organ system pathology, gastrointestinal manifestations are uncommon and may be associated with significant morbidity. We highlight an unusual case of systemic amyloidosis implicated in the formation of a colovesical fistula that required surgical intervention. It is essential to consider amyloidosis as a differential for fistulous formations in the gastrointestinal tract, and such patients should undergo timely endoscopic investigations should they present with gastrointestinal symptoms. Further research is needed to gain an in-depth understanding and formulate management strategies for amyloidosis-related gastrointestinal fistulas.
